# Refining risk stratification for primary prevention ICD therapy: Insights from cardiac MRI in the DO-IT registry

**DOI:** 10.1016/j.hroo.2026.04.013

**Published:** 2026-04-24

**Authors:** Marthe A.J. Becker, Luuk H.G.A. Hopman, Tom E. Verstraelen, Jos Perdeck, Anne-Lotte C.J. van der Lingen, Anton E. Tuinenburg, Lucas V.A. Boersma, Alexander H. Maass, Peter Paul H.M. Delnoy, Stefan A.J. Timmer, Tjeerd Germans, Vokko P. van Halm, Arthur A.M. Wilde, Cornelis P. Allaart, DO-IT investigators

**Affiliations:** 1 Department of Cardiology, Amsterdam UMC, Amsterdam, The Netherlands; 2 Department of Cardiology, University Medical Centre Utrecht, Utrecht, The Netherlands; 3 Cardiology Department, St. Antonius Ziekenhuis Nieuwegein, Nieuwegein, The Netherlands; 4 Department of Cardiology, University Medical Centre Groningen, Groningen, The Netherlands; 5 Department of Cardiology, Isala Klinieken, Zwolle, The Netherlands; 6 Department of Cardiology, Noordwest Ziekenhuisgroep, Alkmaar, The Netherlands

**Keywords:** Primary prevention implantable cardioverter-defibrillator, Cardiac magnetic resonance imaging, Ventricular arrhythmias, Mortality, Ischemic cardiomyopathy, Nonischemic dilated cardiomyopathy

## Abstract

**Background:**

Implantable cardioverter-defibrillators (ICDs) are recommended for the primary prevention of sudden cardiac death in patients with reduced left ventricular ejection fraction (LVEF). However, reliance on LVEF alone may inadequately identify patients at the highest risk.

**Objective:**

This study aimed to evaluate the prognostic value of cardiac magnetic resonance imaging (CMR), particularly late gadolinium enhancement (LGE), in patients with ischemic cardiomyopathy (ICM) and nonischemic dilated cardiomyopathy (NIDCM).

**Methods:**

This multicenter substudy of the Dutch Outcome in ICD Therapy registry included 252 patients with a reduced LVEF (≤35%) who underwent CMR before primary prevention ICD implantation. NIDCM was the underlying etiology in 52% and cardiac resynchronization therapy (CRT) was present in 38%. The median follow-up was 4.3 years. Outcomes were appropriate device therapy (ADT) and all-cause mortality.

**Results:**

LGE was present in 70% and was significantly more extensive in patients with ICM than in those with NIDCM. During follow-up, ADT occurred in 15%, and 19% died, with similar rates between the ICM and NIDCM groups. LGE presence and extent were not associated with ADT or mortality, in the overall cohort nor in subgroup analyses stratified by etiology or non-CRT status. In contrast, right ventricular dysfunction and increased left ventricular end-diastolic volume were associated with outcomes.

**Conclusion:**

LGE on CMR was not associated with ADT or mortality in patients with ICM or NIDCM, nor in the non-CRT subgroup, suggesting a limited role in guiding ICD therapy. In contrast, markers of biventricular dysfunction and adverse remodeling exhibited a prognostic value, highlighting their potential to refine risk stratification beyond traditional LVEF-based criteria.

**Key Findings**

▪ In patients with reduced left ventricular (LV) function owing to ischemic or nonischemic cardiomyopathy who receive guideline-directed therapy, clinical event rates remain low over a 4-year follow-up period.
▪ Contrary to previous reports, this study could not find a significant association between either the presence or the extent of late gadolinium enhancement and device therapy or survival in patients with ischemic or nonischemic dilated cardiomyopathy and primary prevention implantable cardioverter-defibrillator.
▪ The underlying etiology of reduced LV determines characteristic changes on cardiac magnetic resonance imaging, leading to distinct predictors of adverse clinical outcomes.

## Introduction

Implantable cardioverter-defibrillator (ICD) therapy is a cornerstone intervention for reducing mortality in patients at risk of life-threatening ventricular arrhythmias, such as ventricular tachycardia and ventricular fibrillation.^1^^,^^2^ Current clinical guidelines advocate ICD implantation for the primary prevention of sudden cardiac death (SCD) in patients with a left ventricular ejection fraction (LVEF) of <35% and symptomatic heart failure (New York Heart Association [NYHA] functional class II or III) or in patients with an LVEF of <30% and asymptomatic heart failure (NYHA class I).^3^

Despite these established criteria, identifying patients most likely to benefit from prophylactic ICD implantation remains a clinical challenge. Emerging data, particularly in patients with nonischemic dilated cardiomyopathy (NIDCM), have raised questions regarding the survival benefit of primary prevention ICDs in this population.^4^, ^5^, ^6^ Similarly, in ischemic cardiomyopathy (ICM), only a minority of patients with prophylactic ICD will experience an appropriate device intervention.^7^ The majority are exposed to potential procedural complications and device-related risks without deriving substantial benefit.^8^^,^^9^ Moreover, advancements in heart failure management over the past decades, including widespread adoption of optimized medical therapy, have markedly improved both life expectancy and the risk profile for SCD.^10^ These developments call into question the clinical utility of existing ICD indications and highlight the need for better risk stratification strategies to ensure optimal patient selection.

Cardiac magnetic resonance imaging (CMR) has provided new insights into arrhythmic risk beyond traditional measures such as LVEF. Late gadolinium enhancement (LGE), a noninvasive marker of myocardial fibrosis, has been associated with SCD, ventricular arrhythmia, and appropriate ICD interventions in both ICM and NIDCM.^2^^,^^11^, ^12^, ^13^, ^14^ Despite these promising associations, contemporary multicenter data exploring the clinical utility of LGE for refining arrhythmic risk stratification remain limited.

The Dutch Outcome in ICD Therapy (DO-IT) registry offers a unique opportunity to examine the role of CMR in refining risk stratification among patients with reduced ejection fraction heart failure owing to ICM or NIDCM.^15^ The present study aimed to identify high-risk patients within this cohort by integrating CMR-derived markers, including LGE.

## Methods

### Study design and population

This study represents a prespecified subanalysis of the national DO-IT registry. The DO-IT registry, described previously, prospectively enrolled consecutive patients with an American College of Cardiology/American Heart Association/European Society of Cardiology class I indication for primary prevention ICD implantation. Eligibility criteria included symptomatic heart failure with NYHA functional class II or III and an LVEF of ≤35% or asymptomatic heart failure (NYHA functional class I) with an LVEF of ≤30%.^3^^,^^16^ Patients enrolled between September 2014 and May 2016 were included in this national multicenter registry.^15^

In the present substudy, patients from the DO-IT registry were included if they underwent clinically indicated CMR within 1 year before ICD implantation. CMR was not mandated by the registry protocol and was performed at the discretion of the treating physician. Consequently, this cohort represents a subset of the overall DO-IT registry population.

This study adhered to the Declaration of Helsinki principles. A written informed consent was obtained from all participants, and the substudy protocol was approved by the institutional ethics committee of Amsterdam UMC, Vrije Universiteit Amsterdam.

### CMR protocol and analysis

CMR was performed at participating centers using 1.5T and 3.0T whole-body scanners equipped with dedicated phased-array cardiac receiver coils. Standardized imaging protocols included electrocardiogram-gated balanced steady-state free precession cine sequences in 3 long-axis planes and a contiguous short-axis stack covering the entire ventricles during breath-hold at expiration. For patients receiving intravenous gadolinium contrast, LGE imaging was performed 10–15 minutes after administration using T1-weighted inversion recovery sequences with optimized inversion times. Experienced CMR-certified physicians visually assessed the presence and distribution of LGE, which was defined as hyperenhancement visible in 2 perpendicular views or 2 consecutive slices.

Incomplete or poor-quality CMR studies were excluded. Dedicated software (CVI42, Circle Cardiovascular Imaging Inc, Calgary, Canada) was used for postprocessing. Left ventricular (LV) and right ventricular (RV) volumes and function were assessed through semiautomated endocardial delineation on cine short-axis images, with manual corrections as needed. Left atrial volumes were calculated using the area-length method. Global longitudinal strain (GLS) was derived from feature tracking on long-axis cine images. LGE quantification used the full-width-at-half-maximum method,^17^ with results expressed as both grams and percentage of LV mass.

ICM was defined as LV dysfunction attributable to significant coronary artery disease, previous myocardial infarction, or coronary revascularization. In addition, a typical ischemic LGE pattern (subendocardial or transmural in a coronary territory) that was consistent with the degree of LV dysfunction was considered supportive of an ischemic etiology. Limited ischemic-pattern LGE findings without known coronary artery disease were not considered sufficient to reclassify patients as having ICM.

### Outcomes and follow-up

The primary endpoint was appropriate device therapy (ADT), defined as ICD-delivered shock or antitachycardia pacing for ventricular tachycardia or ventricular fibrillation. The secondary endpoint was all-cause mortality. ICD interrogation and clinical follow-up were conducted approximately every 6 months, with follow-up extending until July 2020.

### Statistical analysis

Continuous variables are reported as mean ± standard deviation or median (interquartile range [IQR]), as appropriate. Normality was assessed visually using histograms and quantile-quantile plots. Between-group comparisons were performed using *t* tests or Mann-Whitney U tests for continuous variables and χ^2^ or Mann-Whitney U tests for categorical and ordinal data. Missing data were excluded from the analysis. Given that this study represents a post hoc analysis of a prospective registry, no formal sample size calculation was performed. Therefore, the relatively limited number of outcome events should be considered when interpreting the absence of statistically significant associations.

For the endpoint ADT, death was considered a competing risk. Therefore, cumulative incidence curves were constructed, and competing risk analyses were performed using Fine–Gray subdistribution hazard models. Subdistributional hazard ratios (SHRs) with 95% confidence intervals (CIs) were reported. For survival analyses of the secondary endpoint mortality, Kaplan-Meier curves, with between-group differences assessed using log-rank tests, were used. Hazard ratios (HRs) with 95% CIs were derived using univariable and multivariable Cox regression models. Variables with *P* < .10 in univariable analysis were entered into multivariable analysis. Final selection was done using backward stepwise selection. Statistical significance was set at *P* < .05 for all analyses. Analyses were performed using SPSS software (IBM SPSS Statistics for Windows, version 28.0. Armonk, NY: IBM Corp) and R statistical software version 4.5.1.

## Results

### Patient characteristics

**Figure 1:**
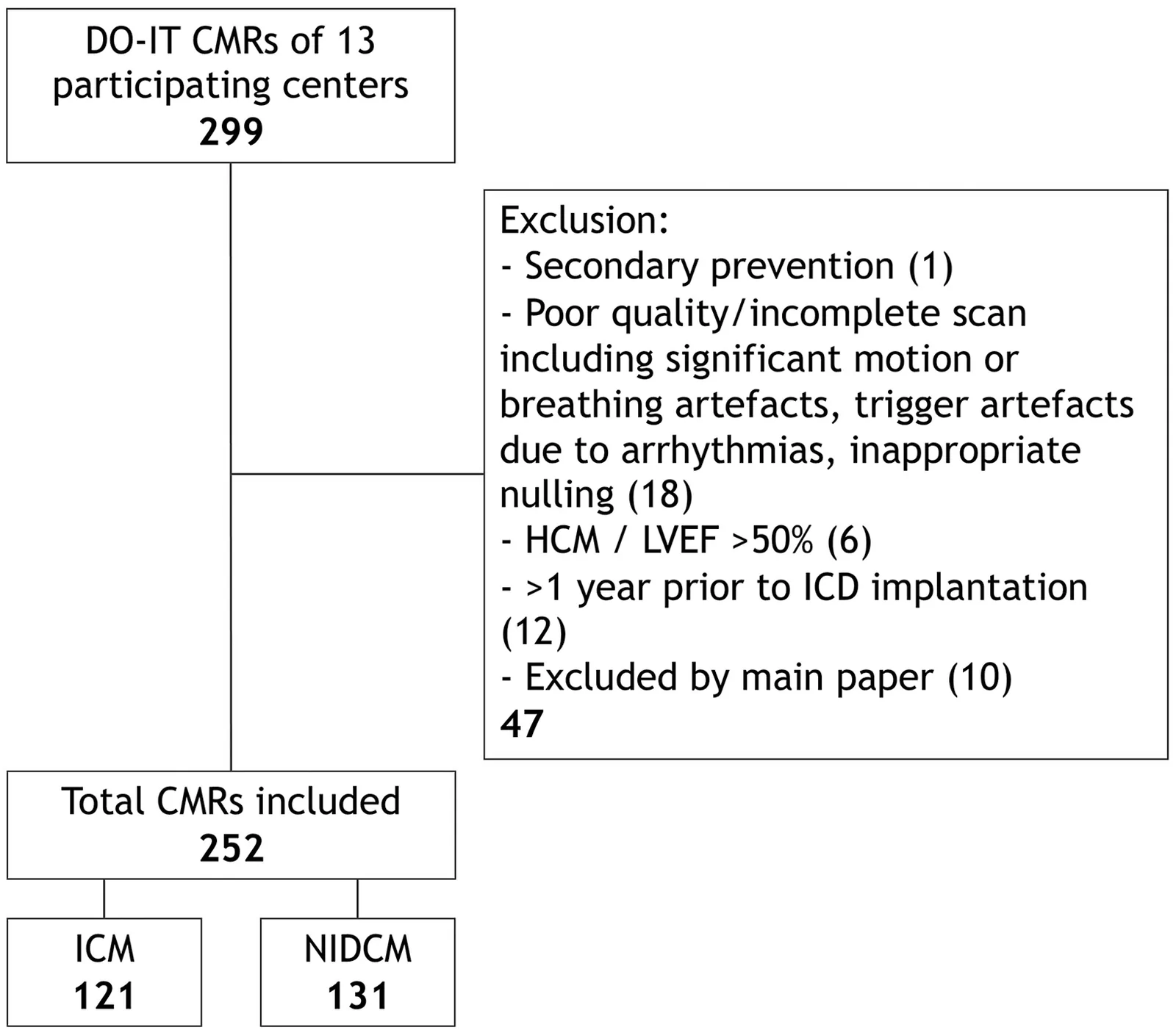
Flowchart demonstrating the selection process of this subgroup analysis of the DO-IT registry. CMR = cardiac magnetic resonance imaging; DO-IT = Dutch Outcome in Implantable Cardioverter-Defibrillator Therapy; HCM = hypertrophic cardiomyopathy; ICD = implantable cardioverter-defibrillator; LVEF = left ventricular ejection fraction.

A total of 299 patients from 13 including centers underwent CMR before ICD implantation. After excluding 47 patients owing to incomplete or poor-quality imaging, an LVEF of >50%, or a scanning-to-implantation interval exceeding 1 year, 252 patients were included in this substudy (Figure 1). The cohort comprised 121 patients (48%) with ICM and 131 patients (52%) with NIDCM.

Baseline characteristics of patients with CMR are presented in Table 1. Compared with patients with ICM, those with NIDCM were younger (61 ± 12 vs 66 ± 9 years; *P* < .001), were less frequently male (55% vs 86%; *P* < .001), and more often received cardiac resynchronization therapy (CRT) (49% vs 26%; *P* < .001). NYHA functional class and the use of guideline-directed medical therapy were similar between groups.

**Table 1:** Baseline characteristics

Characteristic	Total (N = 252)	ICM, n = 121 (48%)	NIDCM, n = 131 (52%)	*P* value
Male sex	176 (70%)	104 (86%)	72 (55%)	<.001
Age (y)	63 ± 11	66 ± 9	61 ± 12	<.001
BMI	27.2 ± 4.6	27.7 ± 4.3	26.7 ± 4.8	.08
NYHA class				.83
I	50 (20%)	25 (21%)	25 (19%)	
II	148 (59%)	69 (57%)	79 (60%)	
III	53 (21%)	27 (22%)	26 (20%)	
Risk factors				
Smoking (current/former)	134/231 (53%)	66/110 (60%)	68/121 (56%)	.56
Hypertension	109/244 (58%)	60/116 (52%)	49/128 (38%)	.04
Hyperlipidemia	95/238 (45%)	64/113 (57%)	31/125 (25%)	<.001
DM	95/250 (40%)	31/120 (26%)	21/130 (16%)	.06
Familial SCD	33/170 (16%)	17/92 (19%)	16/111 (14%)	.44
Medical history				
Infarction	90/249 (36%)	90/120 (75%)	0/129 (0)	<.001
PCI	80/251 (32%)	77/120 (64%)	3/131 (2%)	<.001
CABG	42/252 (17%)	37/121 (31%)	5/131 (4%)	<.001
Atrial fibrillation	64/244 (26%)	33/117 (28%)	31/127 (24%)	.50
Documented NSVT	37/241 (15%)	9/114 (8%)	28/127 (22%)	<.01
Hospitalization for heart failure <1 y	67/248 (27%)	29/119 (24%)	38/129 (30%)	.37
CAG	233 (92%)	117 (97%)	116 (88%)	.03
Coronary artery disease				<.001
Non(significant)	127 (55%)	17 (15%)	105 (91%)	
1VD	34 (15%)	24 (21%)	10 (9%)	
2VD	38 (16%)	38 (32%)	0	
3VD	26 (11%)	26 (22%)	0	
Stenosis in graft	6 (3%)	6 (5%)	0	
Unknown	11 (5%)	10 (9%)	1 (1%)	
Medication				
ACEi/ARB	229 (91%)	104 (86%)	112 (86%)	.92
Beta-blocker	216 (86%)	107 (89%)	122 (93%)	.28
Diuretics	174 (69%)	78 (65%)	96 (73%)	.13
MRA	134 (53%)	58 (48%)	76 (58%)	.11
Device				<.01
S-ICD	25 (10%)	12 (10%)	13 (10%)	
Single-chamber	97 (39%)	56 (46%)	41 (31%)	
Dual-chamber	35 (14%)	22 (18%)	13 (10%)	
CRT-D	95 (38%)	31 (26%)	64 (49%)	

Missing or unknown data were excluded from analysis.

ACEi = angiotensin-converting enzyme inhibitor; ARB = angiotensin receptor blocker; BMI = body mass index; CABG = coronary artery bypass graft; CAG = coronary angiography; CRT-D = cardiac resynchronization therapy defibrillator; DM = diabetes mellitus; ICM = ischemic cardiomyopathy; MRA = mineralocorticoid receptor antagonist; NIDCM = nonischemic dilated cardiomyopathy; NSVT = nonsustained ventricular tachycardia; NYHA = New York Heart Association; PCI = percutaneous coronary intervention; SCD = sudden cardiac death; S-ICD = subcutaneous implantable cardioverter-defibrillator.

### CMR parameters

Key CMR findings are presented in Table 2. The median LVEF in the cohort was 24% (IQR 20–30), and the mean GLS was −6.9% ± 2.6%. RV dysfunction (RV ejection fraction [RVEF] <45%) was present in 46% of patients, with a mean RVEF of 45% ± 13%. LGE imaging was performed in 96% of patients, with LGE detected in 70% (n = 165); the median extent of LGE was 14.1 g (IQR 8.2–24.7).

**Table 2:** Characteristics on LGE-CMR

Characteristic	Total (N = 252)	ICM (n = 121)	NIDCM (n = 131)	*P* value
LV				
LVEDV (mL)	295 ± 72	284 ± 70	306 ± 71	.02
LVEDVi (mL/m^2^)	148 ± 37	140 ± 36	156 ± 36	<.01
LVEF (%)	24 [IQR 20–30]	26 [22–31]	24 [19–27]	.06
LV mass (g)	155 ± 41	150 ± 38	159 ± 43	.08
GLS (%)	−6.9 ± 2.6	−6.9 ± 2.5	−6.6 ± 2.7	.28
RV				
RVEDV (mL)	168 ± 58	160 ± 53	175 ± 61	.04
RVEDVi (mL/m^2^)	84 ± 27	79 ± 25	89 ± 29	<.01
RVEF (%)	45 ± 13	46 ± 13	43 ± 13	.02
RV dysfunction	116 (46%)	43 (36%)	73 (56%)	<.01
LA				
LAVi (mL/m^2^)	56 ± 21	54 ± 19	59 ± 22	.07
LA emptying fraction (%)	39 ± 16	40 ± 16	38 ± 17	.39
LGE				
Gadolinium contrast administration	242 (96%)	114 (94%)	128 (98%)	
LGE presence	165 (70%)	101 (89%)	64 (52%)	<.001
LGE extent (g)	14.1 [8.2–24.7]	18.8 [12.1–29.4]	9.0 [6.0–14.3]	<.001
Ischemic pattern	126 (53%)	95 (84%)	31 (25%)	<.001
Nonischemic pattern	68 (30%)	20 (18%)	48 (39%)	<.001

CMR = cardiac magnetic resonance imaging; GLS = global longitudinal strain; ICM = ischemic cardiomyopathy; IQR = interquartile range; LA = left atrium; LAVi = left atrial volume indexed; LGE = late gadolinium enhancement; LV = left ventricle; LVEDV(i) = left ventricular end-diastolic volume (indexed); LVEF = left ventricular ejection fraction; NIDCM = nonischemic dilated cardiomyopathy; RV = right ventricle; RVEDV(i) = right ventricular end-diastolic volume (indexed); RVEF = right ventricular ejection fraction.

When stratified by etiology, patients with NIDCM exhibited significantly larger LV and RV volumes than those with ICM. The LVEF trended lower in NIDCM than in ICM (*P* = .06), whereas RVEF was significantly lower in NIDCM (mean RVEF 43% ± 13% vs 47% ± 12%; *P* = .02). LGE was more prevalent in ICM than in NIDCM (89% vs 52%; *P* < .001), and the extent of myocardial fibrosis was greater in ICM (median LGE 18.8 g [IQR 12.1–29.4] vs 9.0 g [IQR 6.0–14.3]; *P* < .001).

### Clinical outcomes

**Figure 2:**
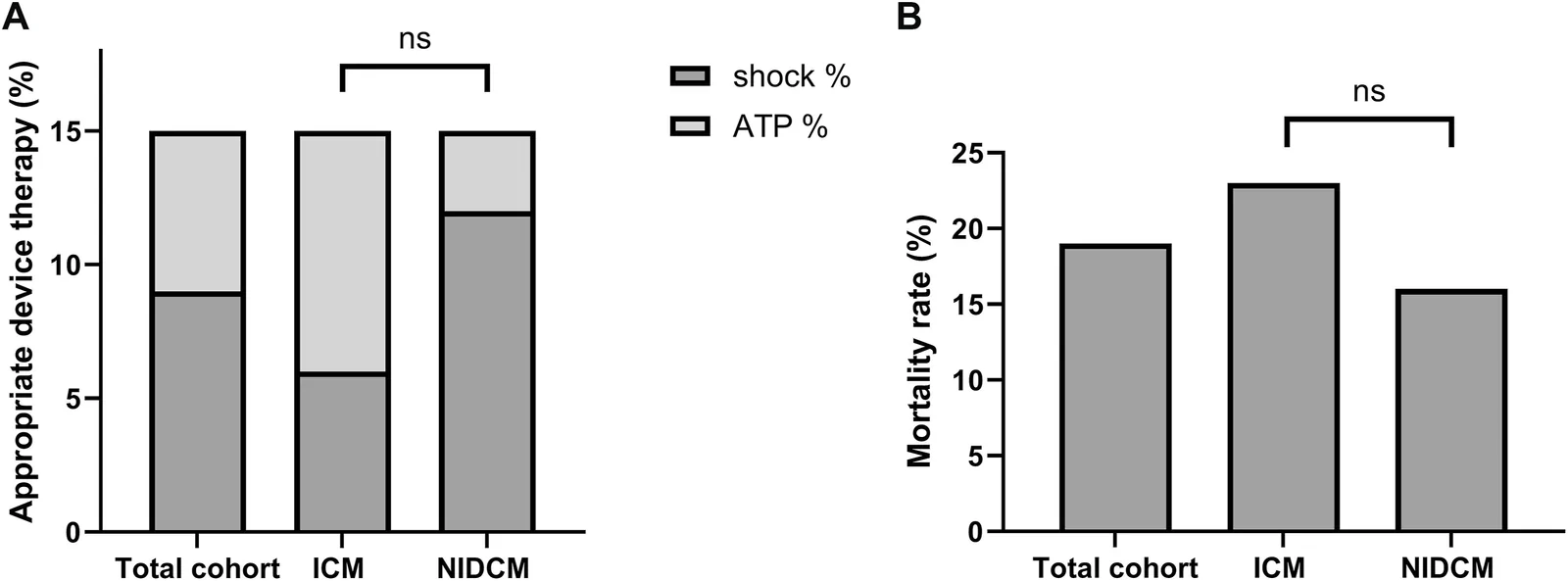
Event rates of primary and secondary endpoints, demonstrating the event rates during follow-up for the primary endpoint appropriate device therapy (**A**) and the secondary endpoint all-cause mortality (**B**) in the total cohort and in ICM and NIDCM. ATP = antitachycardia pacing; ICM = ischemic cardiomyopathy; NIDCM = nonischemic cardiomyopathy; ns = nonsignificant.

**Figure 3:**
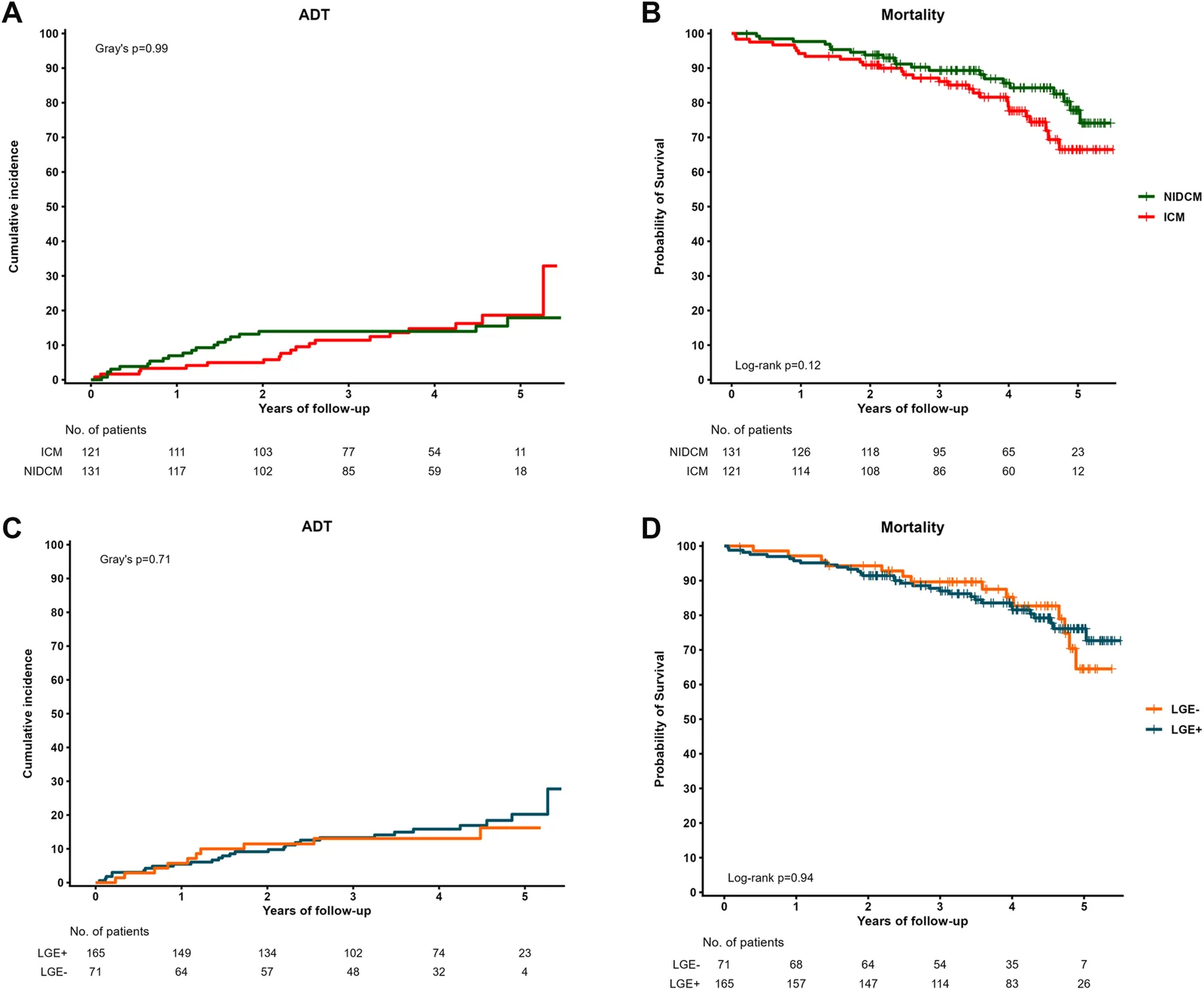
Survival curves for the primary and secondary endpoints. Cumulative incidence function curves of the primary endpoint ADT (**A and C**) and Kaplan-Meier survival curves of the secondary endpoint all-cause mortality (**B and D**), stratified by underlying etiology (**A and B**) and the presence of LGE on CMR (**C and D**). ADT = appropriate device therapy; CMR = cardiac magnetic resonance imaging; ICM = ischemic cardiomyopathy; LGE = late gadolinium enhancement; NIDCM = nonischemic dilated cardiomyopathy

Over a median follow-up of 4.3 years (95% CI 4.1–4.5), 38 patients (15%) experienced ADT, including 22 (9%) who received ICD shocks. ADT rates were similar between ICM and NIDCM (15% vs 15%; *P* = .93) (Figure 2). Mortality occurred in 49 patients (19%), with numerically higher but statistically comparable rates in ICM with NIDCM (23% vs 16%; *P* = .15). Among the 49 deaths, causes were classified as cardiac in 21 cases (11 owing to heart failure, 4 owing to arrhythmia, and 6 owing to other or unspecified cardiac causes), noncardiac in 20 cases, and unknown in 8 cases. Kaplan-Meier analysis revealed no significant difference in overall survival between ICM and NIDCM (*P* = .12) (Figure 3).

### Predictors of ADT and mortality

In the overall cohort, univariable predictors of ADT included male sex (SHR 2.52; *P* = .04), atrial fibrillation (SHR 2.66; *P* < .01), higher LV end-diastolic volume (SHR per 10 mL increase 1.05; *P* < .01), and increased LV mass (SHR 1.01; *P* < .01). Conversely, NYHA class III was inversely associated with ADT (SHR 0.19; *P* = .01). On multivariable analysis, NYHA class I, atrial fibrillation, and LV mass remained independent predictors of ADT (Table 3). Subgroup analyses revealed significant between-group differences in univariable predictors of ADT (Supplemental Table 1). Although not statistically significant, patients with lower LVEF tended toward worse outcomes (Supplemental Figure 1).

**Table 3:** Univariable and multivariable analyses for the occurrence of the primary endpoint appropriate ICD therapy

Variable	Univariable	*P* value	Multivariable	*P* value
	SHR [95% CI]		SHR [95% CI]	
Male sex	2.52 [1.07–5.93]	.04		ns
Age (y)	1.00 [0.97–1.02]	>.9		
ICM	1.00 [0.53–1.86]	>.9		
NYHA class				
I	1.00	Ref	1.00	Ref
II	0.52 [0.26–1.04]	.06	0.52 [0.25–1.07]	.08
III	0.19 [0.05–0.67]	.01	0.12 [0.03–0.52]	<.01
Atrial fibrillation	2.66 [1.42–4.98]	<.01	2.72 [1.43–5.20]	<.01
Documented NSVT	1.63 [0.72–3.70]	.20		
Hospitalization for heart failure <1 y	0.48 [0.20–1.14]	.10		
Familial SCD	0.86 [0.34–2.15]	.70		
CRT-D	0.78 [0.41–1.51]	.50		
CMR				
LVEDV per 10 mL	1.05 [1.01–1.09]	<.01		ns
LVEDVi (mL/m^2^)	1.01 [1.00–1.01]	.08		
LVEF (%)	0.99 [0.95–1.02]	.50		
LV mass (g)	1.01 [1.00–1.02]	<.01	1.01 [1.00–1.01]	.01
GLS (%)	0.98 [0.88–1.09]	.70		
RVEF (%)	1.00 [0.97–1.02]	.70		
RV dysfunction	1.41 [0.76–2.63]	.30		
LAVi (mL/m^2^)	1.01 [1.00–1.03]	.05		ns
LA emptying fraction (%)	0.99 [0.97–1.01]	.30		
LGE presence	1.17 [0.57–2.40]	.70		
LGE extent (g)	1.02 [0.99–1.04]	.20		

ACEi = angiotensin-converting enzyme inhibitor; ARB = angiotensin receptor blocker; BMI = body mass index; CI = confidence interval; CMR = cardiac magnetic resonance imaging; CRT-D = cardiac resynchronization therapy defibrillator; DM = diabetes mellitus; GLS = global longitudinal strain; ICM = ischemic cardiomyopathy; LA = left atrium; LAVi = left atrial volume indexed; LGE = late gadolinium enhancement; LV = left ventricle; LVEDV(i) = left ventricular end-diastolic volume (indexed); LVEF = left ventricular ejection fraction; NSVT = nonsustained ventricular tachycardia; NYHA = New York Heart Association; Ref = reference; RV = right ventricle; RVEF = right ventricular ejection fraction; SCD = sudden cardiac death; SHR = subdistributional hazard ratio.

Neither the presence nor extent of LGE was associated with ADT in the overall cohort or within the ICM and NIDCM subgroups (*P* > .05 for all comparisons). There was no significant association between etiology and ADT (mean SHR 1.00; *P* > .90 for ICM). Similarly, when assessing ICD recipients without CRT, no significant association was observed between LGE and ADT (*P* = .8), including within the NIDCM subgroup (*P* > .9) (Supplemental Figure 2). CMR variables univariably associated with ICD shocks included increased cardiac volumes, RV dysfunction, and LV mass, primarily driven by findings in the ICM subgroup. In NIDCM, increased LV end-diastolic volume was associated with ICD shock (Supplemental Table 2).

CRT was not associated with ADT in the overall cohort (SHR 0.78; *P* = .5). In the NIDCM subgroup, ADT was numerically less frequent in patients with CRT than in those without CRT (11% vs 19%; *P* = .18), although this did not reach statistical significance.

For mortality, significant univariable predictors in the overall cohort included age (HR 1.06 per year; *P* < .01), NYHA class III (HR 2.46; *P* = .05), LVEF (HR 0.96 per 1% increase; *P* = .05), impaired GLS (HR 1.21 per 1% decrease; *P* < .01), RVEF (HR 0.97 per 1% increase; *P* = .02), and reduced left atrial emptying fraction (HR 0.98 per 1% increase; *P* = .04). Neither the presence nor the extent of LGE was associated with mortality. Subgroup analyses revealed distinct predictors in ICM and NIDCM (Supplemental Table 3). CRT was not significantly associated with mortality in the overall cohort (HR 0.60; *P* = .29) or in the NIDCM subgroup (HR 0.54; *P* = .19).

## Discussion

This study assessed the prognostic value of CMR parameters, including LGE, in patients undergoing primary prevention ICD implantation. The findings highlight the complexities of risk stratification in this heart failure population, given that LGE, proposed as a key risk marker, was not associated with outcomes in the total cohort, nor in the ICM and NIDCM subgroups.

Eligibility for primary prevention ICD implantation is primarily determined by LVEF.^18^ However, the limitations of using LVEF as the sole criterion have become increasingly apparent, particularly given advances in heart failure therapies over the past decade. These therapies have significantly improved survival and reduced the incidence of SCD.^10^^,^^19^, ^20^, ^21^ As a result, a considerable proportion of patients who receive an ICD for primary prevention never experience ADT, and the incidence of inappropriate shocks often equals or exceeds that of appropriate interventions.^22^ In addition, complications related to ICD implantation including infection, lead dislodgement, and device malfunction raise concerns regarding the clinical and cost-effectiveness of the current ICD strategy.^8^^,^^9^ The low event rates of ADT and mortality in the present study are consistent with contemporary reports demonstrating improved outcomes in patients with heart failure,^10^^,^^20^^,^^22^ in contrast to older studies that reported significantly higher event rates and mortality,^20^ underscoring the evolving risk profile of this population. Although the sample size and event rate were modest, LVEF was not associated with ADT in this cohort, suggesting that LVEF alone may no longer provide adequate risk stratification for SCD.

Previous studies have linked the presence and extent of LGE to an increased risk of SCD and adverse cardiac outcomes.^14^^,^^23^ However, in this cohort, neither the presence nor the extent of LGE was significantly associated with ADT or all-cause mortality, both in the overall population and in the ICM and NIDCM subgroups. Moreover, in patients with NIDCM without CRT, LGE was not associated with outcomes. These findings challenge the emerging consensus supporting LGE-based risk stratification for ICD decision making.^5^ Notably, LV mass, RV dysfunction, and increased LV dilation emerged as stronger predictors of adverse outcomes, emphasizing the prognostic significance of markers of advanced disease progression and remodeling. These observations suggest that future risk stratification strategies should incorporate comprehensive assessments of cardiac remodeling and biventricular function to improve patient selection for ICD therapy.

In addition to CMR variables, several clinical parameters were associated with outcomes. Patients with NYHA functional class I had a higher risk of ADT, consistent with previous findings,^24^ whereas those with NYHA class III symptoms exhibited increased mortality, reflecting distinct pathophysiological trajectories of arrhythmic vs pump failure–related death. Notably, a substantial proportion of patients, particularly those with NIDCM, received a CRT defibrillator; however, the presence of CRT defibrillator was not associated with reduced mortality or ADT. This finding contrasts with previous studies demonstrating a survival benefit of CRT in appropriately selected patients with heart failure.^25^ Moreover, recent national guidelines recommend CRT without a defibrillator in patients with NIDCM, no significant LGE, and no identifiable genetic etiology.^5^ In the present study, a trend toward lower ADT rates was observed in the NIDCM subgroup with CRT, although this did not reach statistical significance. The absence of a significant association may reflect selection bias, limited sample size, or competing risks, including advanced age, frailty, and comorbid conditions that influence survival. Hence, more research on SCD risk in this specific patient population is necessary to assess the potential effect of the current national guideline change on CRT pacemakers.

The overall improvement in outcomes among patients with heart failure receiving optimal medical therapy, along with the inconsistencies in prognostic markers noted earlier, highlights the need for further investigation, ideally using prospective data.^26^ Several ongoing clinical trials aim to refine risk stratification in this primary prevention ICD population. Currently, clinical trials including CMR-ICD-DZHK23 (NCT04558723), BRITISH (NCT05568069), SPANISH-1 (NCT06055504), the DUTCH-ICD trial, and the PROFID (NCT05665608) are evaluating the role of CMR-derived fibrotic markers in guiding ICD implantation, underscoring the increasing interest in integrating advanced imaging biomarkers into clinical decision making. However, the present findings suggest caution in broadly applying LGE as the key risk marker across all patient subsets. Given the low incidence of arrhythmic events in heart failure and the modest HRs of SCD predictors observed in this study, the clinical relevance of prophylactic ICD implantation warrants careful reconsideration. Future studies should explore the integration of advanced imaging biomarkers, including RV function and volumetric parameters, with clinical and electrophysiological data to develop a more comprehensive and individualized approach to ICD therapy.

### Limitations

This study has several limitations that should be considered. First, the heterogeneity of the patient population, encompassing both ischemic and nonischemic etiologies, may affect generalizability. The total cohort size and size of both subgroups may have limited statistical power to detect subtle associations between LGE and clinical outcomes. Second, the limited number of events reduces statistical power and increases the possibility of type II error, particularly in subgroup analyses. CMR was performed at the discretion of the treating physicians, which may have introduced selection bias for the present cohort. Moreover, underlying genetic cardiomyopathies, particularly in NIDCM, were not routinely assessed and may have influenced arrhythmic risk. Third, advanced tissue characterization techniques such as T1 mapping and extracellular volume fraction quantification, which have demonstrated potential in risk stratification, were not available in this cohort. Fourth, variations in LGE quantification techniques across studies may contribute to inconsistencies in the reported prognostic utility of LGE. Finally, the moderate follow-up period and low event rates, particularly among patients on optimized medical therapy, may have constrained our ability to capture long-term outcomes.

## Conclusion

This study underscores the limitations of traditional risk stratification approaches in heart failure patients with reduced LVEF undergoing primary prevention ICD implantation. Although CMR-derived LGE has shown promise in previous studies, its utility as a universal risk marker for SCD was not corroborated in our cohort. Instead, markers of advanced disease progression, including biventricular dysfunction and remodeling, may offer greater prognostic value. These findings highlight the need for ongoing research to refine risk stratification and optimize ICD implantation strategies in contemporary heart failure populations.

## Disclosures

The authors have no conflicts of interest to disclose.
