# Supplementary material for: Refining risk stratification for primary prevention ICD therapy: Insights from cardiac MRI in the DO-IT registry
Source: Heart Rhythm O2. 2026 Apr 24;7(8):1546–54. doi: 10.1016/j.hroo.2026.04.013 (PMC13523596; doi:10.1016/j.hroo.2026.04.013)
Supplement: Supplementary file 1 [file mmc1.docx]

SUPPELEMENTS

- TABLE S1. Univariable predictors of ADT in ICM and NIDCM
- TABLE S2. Univariabel predictors of the occurrence of shock
- TABLE S3. Univariable predictors of the secondary endpoint all-cause mortality in the total cohort and in ICM and NIDCM
- Figure S1
- Figure S2
- List of DO-IT investigators

TABLE S1. Univariable predictors of ADT in ICM and NIDCM

|  | **ICM** |  | **NIDCM** |  |
| --- | --- | --- | --- | --- |
|  | **Univariable** |  | **Univariable** |  |
|  | **SHR [95% CI]** | **P-value** | **SHR [95% CI]** | **P-value** |
| Male sex | 3.06 [0.44-21.10] | 0.30 | 2.68 [0.99-7.24] | 0.05 |
| Age (years) | 0.98 [0.93-1.04] | 0.50 | 1.01 [0.98-1.04] | 0.70 |
| NYHA class |  |  |  |  |
| I | ref |  | ref |  |
| II | 1.18 [0.37-3.72] | 0.80 | 0.28 [0.12-0.68] | <0.01 |
| III | 0.42 [0.07-2.45] | 0.30 | 0.09 [0.01-0.69] | 0.02 |
| Atrial fibrillation | 2.27 [0.92-5.58] | 0.07 | 3.20 [1.36-7.53] | <0.01 |
| Documenten NSVT | 3.36 [0.89-12.60] | 0.07 | 1.23 [0.43-3.47] | 0.70 |
| HF hospitalization <1y | 0.56 [0.16-1.94] | 0.40 | 0.40 [0.12-1.33] | 0.14 |
| Familial SCD | 0.88 [0.27-2.95] | 0.80 | 0.78 [0.19-3.30] | 0.70 |
| **CRT-D** | 1.19 [0.48-2.99] | 0.70 | 0.54 [0.22-1.34] | 0.20 |
|  |  |  |  |  |
| **CMR** |  |  |  |  |
| LVEDV per 10mL | 1.05 [1.00-1.10] | 0.05 | 1.05 [1.00-1.11] | 0.07 |
| LVEDVi (ml/m^2^) | 1.01 [1.00-1.02] | 0.09 | 1.00 [0.99-1.02] | 0.40 |
| LVEF (%) | 0.98 [0.93-1.03] | 0.40 | 1.00 [0.95-1.05] | 0.90 |
| LV mass (g) | 1.02 [1.00-1.03] | <0.01 | 1.00 [1.00-1.01] | 0.30 |
| GLS (%) | 0.93 [0.78-1.12] | 0.50 | 1.01 [0.89-1.14] | 0.90 |
| RVEF (%) | 1.00 [0.96-1.04] | >0.9 | 0.99 [0.97-1.02] | 0.70 |
| RV dysfunction | 1.66 [0.67-4.08] | 0.30 | 1.223[0.51-3.00] | 0.60 |
| LAVi (ml/m^2^) | 1.02 [0.99-1.05] | 0.14 | 1.01 [0.99-1.03] | 0.20 |
| LA emptying fraction (%) | 1.00 [0.97-1.02] | 0.80 | 0.99 [0.96-1.01] | 0.20 |
| LGE presence | 2.17 [0.31-16.50] | 0.40 | 0.99 [0.41-2.41] | >0.9 |
| LGE extent (g) | 1.02 [0.99-1.05] | 0.20 | 1.03 [0.98-1.07] | 0.20 |

TABLE S2. Univariabel predictors of the occurrence of shock

|  | **Total** |  | **ICM** |  | **DCM** |  |
| --- | --- | --- | --- | --- | --- | --- |
|  | **Univariable** |  | **Univariable** |  | **Univariable** |  |
|  | **SHR [95% CI]** | **P-value** | **SHR [95% CI]** | **P-value** | **SHR [95% CI]** | **P-value** |
| Male sex | 2.84 [0.85 – 9.50] | 0.09 | * | ns | 3.52 [1.00 - 12.32] | 0.05 |
| Age (years) | 1.00 [0.96 – 1.03] | 0.81 | 0.98 [0.88 – 1.08] | 0.66 | 1.01 [0.98 – 1.05] | 0.49 |
| NYHA functional class |  |  |  |  |  |  |
| I | 1.00 | ref | 1.00 | ref | 1.00 | ref |
| II | 0.38 [0.16 – 0.93] | 0.03 | 0.36 [0.07 – 1.81] | 0.21 | 0.36 [0.13 – 1.04] | 0.06 |
| III | 0.19 [0.04 – 0.90] | 0.04 | 0.31 [0.03 – 2.97] | 0.31 | 0.14 [0.02 – 1.18] | 0.07 |
| AF | 4.19 [1.80 – 9.75] | <0.001 | 3.48 [0.78 – 15.48] | 0.1 | 4.83 [1.75 – 13.36] | <0.01 |
| Documented NSVT | 2.26 [0.87 – 5.90] | 0.09 | 11.07 [2.57 – 47.77] | <0.01 | 0.90 [0.24 – 3.32] | 0.85 |
| HF hospitalization <1y | 0.26 [0.06 – 1.09] | 0.07 | 0.50 [0.06 – 3.79] | 0.51 | 0.16 [0.02 – 1.19] | 0.07 |
| Familial SCD | 0.58 [0.14 – 2.48] | 0.46 | * |  | 1.15 [0.26 – 5.05] | 0.85 |
| **CRT** | 0.61 [0.24 – 1.54] | 0.29 | 0.48 [0.06 – 3.79] | 0.51 | 0.51 [0.18 – 1.48] | 0.22 |
| **CMR** |  |  |  |  |  |  |
| LVEDV per 10mL | 1.09 [1.04 – 1.13] | <0.001 | 1.09 [1.03 – 1.17] | <0.01 | 1.08 [1.02 – 1.14] | 0.01 |
| LVEDVi | 1.01 [1.00 – 1.02] | <0.01 | 1.01 [1.00 – 1.02] | <0.01 | 1.01 [0.99 – 1.02] | 0.24 |
| LVEF | 0.96 [0.92 – 1.01] | 0.15 | 0.95 [0.87 – 1.04] | 0.27 | 0.98 [0.92 – 1.04] | 0.48 |
| LV mass | 1.01 [1.00 – 1.02] | <0.01 | 1.02 [1.00 – 1.04] | 0.07 | 1.01 [1.00 – 1.02] | 0.09 |
| GLS | 0.95 [0.83 – 1.09] | 0.47 | 0.91 [0.70 – 1.18] | 0.48 | 0.99 [0.86 – 1.14] | 0.84 |
| RVEF | 0.97 [0.94 – 1.00] | 0.04 | 0.94 [0.90 – 0.99] | 0.02 | 0.99 [0.96 – 1.02] | 0.54 |
| RV dysfunction | 2.62 [1.07 – 6.38] | 0.03 | 4.84 [0.96 – 24.49] | 0.06 | 1.64 [0.57 – 4.75] | 0.36 |
| LAVi | 1.02 [1.01 – 1.04] | <0.01 | 1.04 [1.00 – 1.08] | 0.03 | 1.01 [0.99 – 1.03] | 0.17 |
| LA emptying fraction | 0.98 [0.96 – 1.01] | 0.15 | 0.99 [0.95 – 1.02] | 0.53 | 0.98 [0.96 – 1.01] | 0.24 |
| LGE presence | 0.84 [0.34 – 2.08] | 0.71 | * |  | 0.89 [0.32 – 2.49] | 0.82 |
| LGE extent | 1.01 [0.97 – 1.06] | 0.63 | 1.02 [0.96 – 1.09] | 0.57 | 1.02 [0.98 – 1.07] | 0.31 |

* inappropriate value due to small cell count

TABLE S3. Univariable analysis of predictors of the secondary endpoint all-cause mortality in the total cohort and in ICM and NIDCM

|  |  |  | **ICM** |  | **NIDCM** |  |
| --- | --- | --- | --- | --- | --- | --- |
|  | **Univariable** |  | **Univariable** |  | **Univariable** |  |
|  | **HR [95% CI]** | **P-value** | **HR [95% CI]** | **P-value** | **HR [95% CI]** | **P-value** |
| Male sex | 1.63 [0.83-3.19] | 0.16 | 0.80 [0.31-2.11] | 0.66 | 2.18 [0.85-5.62] | 0.11 |
| Age (years) | 1.06 [1.03-1.10] | <0.01 | 1.09 [1.03-1.14] | <0.01 | 1.04 [0.99-1.08] | 0.12 |
| Ischaemic | 1.57 [0.89-2.77] | 0.12 |  |  |  |  |
| NYHA class |  | 0.04 |  | 0.08 |  | 0.29 |
| I | 1.00 | ref | 1.00 | ref | 1.00 | ref |
| II | 1.16 [0.50-2.68] | 0.73 | 1.99 [0.57-6.87] | 0.28 | 0.66 [0.20-2.11] | 0.48 |
| III | 2.46 [1.01-5.98] | 0.05 | 3.84 [1.06-13.99] | 0.04 | 1.47 [0.42-5.23] | 055 |
| Atrial fibrillation | 1.94 [1.09-3.45] | 0.03 | 3.39 [1.61-7.13] | <0.01 | 0.86 [0.31-2.39] | 0.78 |
| Documented NSVT | 0.67 [0.27-1.71] | 0.41 | 2.34 [0.79-7.85] | 0.17 | 0.33 [0.08-1.44] | 0.14 |
| Hospitalization for heart failure <1 year | 1.46 [0.80-2.65] | 0.22 | 1.59 [0.72-3.52] | 0.25 | 1.36 [0.55-3.37] | 0.51 |
| Familial SCD | 1.63 [0.74-3.61] | 0.23 | 1.07 [0.35-3.25] | 0.90 | 2.32 [0.74-7.22] | 0.15 |
| CRT-D | 1.01 [0.57-1.80] | 0.97 | 1.10 [0.48-2.50] | 0.82 | 1.18 [0.50-2.79] | 0.70 |
| **CMR** |  |  |  |  |  |  |
| LVEDV per 10mL | 1.02 [0.98-1.05] | 0.40 | 0.99 [0.94-1.05] | 0.81 | 1.05 [1.00-1.10] | 0.07 |
| LVEDVi (ml/m^2^) | 1.01 [0.99-1.01] | 0.09 | 1.00 [0.99-1.01] | 0.51 | 1.01 [1.00-1.03] | 0.01 |
| LVEF (%) | 0.96 [0.93-0.99] | 0.05 | 0.95 [0.90-1.00] | 0.05 | 0.96 [0.91-1.02] | 0.22 |
| LV mass (g) | 1.00 [0.99-1.01] | 0.83 | 1.00 [0.99-1.01] | 0.52 | 1.01 [1.00-1.02] | 0.21 |
| GLS (%) | 1.21 [1.08-1.36] | <0.01 | 1.37 [1.16-1.61] | <0.001 | 1.13 [0.96-1.33] | 0.16 |
| RVEF (%) | 0.97 [0.95-0.99] | 0.02 | 0.95 [0.92-0.97] | 0.02 | 1.00 [0.97-1.04] | 0.91 |
| RV dysfunction | 1.93 [1.09-3.42] | 0.24 | 3.18 [1.50-6.75]] | <0.01 | 1.36 [0.56-3.29] | 0.49 |
| LAVi (ml/m^2^) | 1.01 [1.00-1.03] | 0.05 | 1.02 [1.01-1.04] | 0.01 | 1.01 [0.99-1.03] | 0.39 |
| LA emptying fraction (%) | 0.98 [0.97-0.99] | 0.04 | 0.96 [0.94-0.99] | <0.01 | 1.00 [0.98-1.03] | 0.94 |
| LGE presence | 0.97 [0.52-1.83] | 0.94 | 0.95 [0.28-3.17] | 0.93 | 0.71 [0.29-1.72] | 0.45 |
| LGE extent (g) | 1.01 [0.99-1.04] | 0.30 | 1.01 [0.98-1.04] | 0.51 | 1.00 [0.92-1.08] | 0.93 |

Abbreviations: ACEi, angiotensin-converting enzyme inhibitors; ARB, angiotensin receptor blockers ; BMI, body mass index; CI, confidence interval; CMR, cardiac magnetic resonance imaging; CRT-D, cardiac resynchronization therapy defibrillator; DM, diabetes mellitus; GLS, global longitudinal strain; HR, hazard ratio; ICM, ischaemic cardiomyopathy; LA left atrium; LAVi, Left atrial volume indexed; LGE, late gadolinium enhancement; LV, left ventricle; LVEDV(i), left ventricular end-diastolic volume (indexed); LVEF, left ventricular ejection fraction; NSVT, non-sustained ventricular tachycardia; NYHA, New York Heart Association function class; RV, right ventricle; RVEF right ventricular ejection fraction. SCD, sudden cardiac death

Figure S1

Survival curves of the primary endpoint appropriate device therapy (A) and secondary endpoint all-cause mortality (B) stratified by LVEF.

Figure S2

Cumulative Incidence Function curve of the primary endpoint ADT in patients without CRT in the total cohort (A) and in the NIDCM subgroup (B).

List of DO-IT investigators

Listed in alphabetical order by institution.

Academic Medical Centre (A.A.M Wilde); Albert Schweitzer Hospital (M.W.F. van Gent); Amphia Hospital (S. Strikwerda); Canisius-Wilhelmina Hospital (L.H.R. Bouwels); Catharina Hospital (P.H. van der Voort); Erasmus Medical Centre (D.A.M.J. Theuns); Flevoziekenhuis (N.R. Bijsterveld); Isala Klinieken (P.P.H.M. Delnoy); Haga Hospital (R. Abels); Kennemer Gasthuis (R. Tukkie); Leiden University Medical Centre (L. van Erven); Maasstad Hospital (M. Firouzi); Maastricht University Medical Centre (K. Vernooy); Martini Hospital (L.H. Takens); Haaglanden Medical Centre (R.W. Grauss); Medical Centre Alkmaar (G.P. Kimman); Medical Centre Leeuwarden (A.E. Borger van der Burg); Medisch Spectrum Twente (M.F. Scholten); Onze Lieve Vrouwe Gasthuis (M. Khan); Rijnstate Hospital (R. Derksen); Scheper Hospital (M.W. Vet); St. Antonius Hospital (L.V.A. Boersma); Tweesteden Hospital (J.W.M.G. Widdershoven); University Medical Centre Groningen (A.H. Maass); Radboud Universiy Medical Centre (M.A. Brouwer); University Medical Centre Utrecht (A.E. Tuinenburg); Vlietland Hospital (H.A.M. Spierenburg); VU University Medical Centre (C.P. Allaart).
